# Antenatal exposure of maternal secondhand smoke (SHS) increases fetal lung expression of RAGE and induces RAGE-mediated pulmonary inflammation

**DOI:** 10.1186/s12931-014-0129-7

**Published:** 2014-10-23

**Authors:** Duane R Winden, David B Barton, Bryce C Betteridge, Jared S Bodine, Cameron M Jones, Geraldine D Rogers, Michael Chavarria, Alex J Wright, Zac R Jergensen, Felix R Jimenez, Paul R Reynolds

**Affiliations:** Department of Physiology and Developmental Biology, Brigham Young University, 375A Widtsoe Building, Provo, UT 84602 USA

**Keywords:** RAGE, Tobacco, Lung, Collagen

## Abstract

**Background:**

Receptors for advanced glycation end-products (RAGE) are immunoglobulin-like pattern recognition receptors abundantly localized to lung epithelium. Our research demonstrated that primary tobacco smoke exposure increases RAGE expression and that RAGE partly mediates pro-inflammatory signaling during exposure. However, the degree to which RAGE influences developing lungs when gestating mice are exposed to secondhand smoke (SHS) has not been determined to date.

**Methods:**

Timed pregnant RAGE null and wild type control mice were exposed to 4 consecutive days of SHS from embryonic day (E) 14.5 through E18.5 using a state of the art nose-only smoke exposure system (Scireq, Montreal, Canada). RAGE expression was assessed using immunofluorescence, immunoblotting, and quantitative RT-PCR. TUNEL immunostaining and blotting for caspase-3 were performed to evaluate effects on cell turnover. Matrix abnormalities were discerned by quantifying collagen IV and MMP-9, a matrix metalloprotease capable of degrading basement membranes. Lastly, TNF-α and IL-1β levels were assessed in order to determine inflammatory status in the developing lung.

**Results:**

Pulmonary RAGE expression was elevated in both dams exposed to SHS and in fetuses gestating within mothers exposed to SHS. Fetal weight, a measure of organismal health, was decreased in SHS-exposed pups, but unchanged in SHS-exposed RAGE null mice. TUNEL assessments suggested a shift toward pulmonary cell apoptosis and matrix in SHS-exposed pups was diminished as revealed by decreased collagen IV and increased MMP-9 expression. Furthermore, SHS-exposed RAGE null mice expressed less TNF-α and IL-1β when compared to SHS-exposed controls.

**Conclusions:**

RAGE augmentation in developing pups exposed to maternal SHS weakens matrix deposition and influences lung inflammation.

## Background

Lung development involves precisely programmed events wherein communication between endoderm and the surrounding mesoderm coordinates cell commitment and differentiation [1]. As development concludes, a vast surface area of respiratory epithelium is positioned opposite a dynamic basement membrane through which gases pass to and from a considerable vascular network. An environment conducive to the appropriate spatial and temporal expression of target genes makes the coordination of specific gene programs possible. Such programs result in the deposition of respiratory tissues critically necessary for terrestrial life.

The receptor for advanced glycation end products (RAGE) is a cell-surface membrane protein of the immunoglobulin superfamily composed of three domains: an extracellular ligand binding domain, a domain necessary for membrane docking, and a cytosolic domain essential in the perpetuation of intracellular signaling events [2]. RAGE is expressed in several organs, but basal expression is primarily observed in the lung [3]. In fact, most other organs known to express RAGE and its signaling intermediates are those in a diseased state [4]. While its role in development is less understood, RAGE may function in discrete ways during the programming of squamous epithelium that must spread and appropriately adhere to matrix substrates [5]. For example, RAGE is identified to the baso-lateral membrane of alveolar epithelial cells and localization in this domain enhances the binding of these epithelial cells to collagen in the matrix [5]. Combined with its role in the establishment of organ architecture, RAGE may also influence organogenesis via its involvement in apoptotic pathways intricately associated with defining cell populations in the mature alveolus [6].

While RAGE expression during lung organogenesis may assist in defining the respiratory compartment, its participation in lung inflammatory signaling may further explain developmental abnormalities. RAGE binds advanced glycation end-products (AGEs) during the orchestration of inflammation and AGEs are commonly detected in tobacco smoke [7]; however, the impact of elevated receptor availability during embryogenesis has not been clearly tested. Additional RAGE ligands including cytokine-like mediators of the S100/calgranulin family and high mobility group box 1 (HMGB-1) [2,8] further implicate downstream signaling pathways potentially involved in mechanisms of abnormal lung derivation. Examples of deleterious effectors to cell turnover and differentiation include Ras and NF-kB [9,10], two factors discovered to be RAGE targets. Because these signaling molecules increase in cases of elevated apoptosis and matrix resorption, RAGE may perpetuate a signaling axis wherein embryonic tissue loss and irreversible parenchymal remodeling occur.

Tobacco smoking and exposure to secondhand smoke (SHS) are widely viewed to be causative factors for childhood asthma and chronic obstructive pulmonary disease (COPD) affecting nearly 3 billion people worldwide [11]. Seminal research by Tager *et al.* initially showed that SHS affected fetal lung development in a landmark study of the effects of smoke exposure on neonatal pulmonary function [12]. Subsequent reasoning led to the concept that antenatal factors could affect normal lung development and that chronic diseases have their origins *in utero*. Short and long term effects of fetal exposure to maternal smoking during gestation results in hypoplastic lungs with fewer air saccules coincident with bronchopulmonary dysplasia (BPD), persistently reduced pulmonary function, and increased incidence and lifelong pulmonary disease. In fact, significant suppression of alveolarization in severe cases of SHS exposure causes neonatal lethality. It is important to emphasize that the main effects of *in utero* SHS exposure on lung growth and differentiation are likely the result of specific alterations in late fetal lung development.

In the current study, the expression dynamics of RAGE were evaluated in the context of SHS exposure and RAGE availability. The current research suggests that RAGE signaling causes deterioration of the alveolar basement membrane through MMP-9 mediated collagen IV destruction, and that RAGE-mediated inflammation observed during SHS exposure may influence the trajectory of pulmonary morphogenesis.

## Methods

### Animals and SHS exposure

All mice were in a C57Bl/6 background. Time mated mice were obtained and embryonic (E) day 0 was noted as the day a vaginal plug was discovered. Pregnant mice were exposed to secondhand smoke (SHS) at the start of the pseudoglandular period of lung development (E14.5) and the fourth consecutive day of SHS exposure was E17.5. Dams were then sacrificed on E18.5, pups were weighed, and lungs were resected for histology or molecular characterization. Mice were housed in a conventional animal facility supplied with pelleted food and water *ad libitum* and maintained on a 12-hour light–dark cycle. Mice were placed in soft restraints and connected to the exposure tower. Animals were exposed to SHS generated by six standard research cigarettes (2R1, University of Kentucky, Lexington, KY) through their noses using a nose-only exposure system (InExpose System, Scireq, Canada). A computer-controlled puff of sidestream smoke was generated for 10 minutes followed by 10 minutes of non-exposure. This process was repeated two additional times for a total of 30 minutes of secondhand smoke exposure per day. The SHS-exposed group inhaled SHS from six consecutive cigarettes per day for four days. The SHS challenge chosen in the present study was associated with a good tolerance of mice to the SHS sessions, and an acceptable level of particulate density concentration according to literature [13,14]. Control animals were restrained similarly and were exposed to room air for the same duration. Animal use was in accordance with IACUC protocols approved by Brigham Young University.

### Lung morphology and immunohistochemistry

Lungs were fixed in 4% paraformaldehyde, embedded in paraffin, and 5 μm sections were obtained. Sections were dehydrated, deparaffinized, and antigen retrieval was performed using the citrate buffer method [15,16]. RAGE immunofluorescence was completed using goat polyclonal IgG (AF1145, 1:500, R&D Systems, Minneapolis, MN). Sections were blocked in 5% donkey serum in PBS for 2 hours at room temperature, followed by incubation with primary antibodies at 4°C overnight. Control sections were incubated in blocking serum alone. After overnight incubation, all sections (including the controls) were washed using PBS/triton prior to the application of Alexa Fluor® 488 Rabbit Anti-Goat IgG (Invitrogen, Carlsbad, CA) secondary antibodies for 1 hour at room temperature. For immunohistochemistry, slides were blocked, incubated with primary and appropriate secondary antibodies that utilize HRP conjugation with the Vector Elite Kit (Vector Laboratories; Burlingame, CA). Antibodies included collagen IV (1:500, Abcam, Cambridge, MA, ab6586) and MMP-9 (1:200, Santa Cruz Biotechnology, Santa Cruz, CA, sc-6840). The TdT-FragEL DNA Fragmentation Detection Kit (Calbiochem, Rockland, MA) was used to immunohistochemically evaluate apoptosis. No staining was observed in sections without primary or secondary antibody.

### Immunoblotting

Lungs from E18.5 mouse embryos were homogenized in RIPA buffer with protease inhibitors (Thermo Fisher). BCA quantification was performed to ensure equal sample concentrations (Thermo Fisher) and Ponceau S staining of transferred membranes was performed to visualize equal loading (not shown). Immunoblotting was performed using antibodies against RAGE (AF1145), collagen IV (Abcam, 1:5,000, ab6586), MMP-9 (Santa Cruz, 1:500, sc-6840) and caspase-3 (Cell Signaling, Beverly, MA, 1:1000, #9662) using standard protocols discussed in previous work [9,17]. Goat anti-rabbit (Vector Labs, Burlingame, CA, PI-1000) secondary antibody concentration was 1:10,000 for collagen IV and 1:5,000 for all other blots. To determine loading consistencies, each membrane was stripped and reprobed with an antibody against mouse beta-actin (dilution 1:1000; Sigma Aldrich, St. Louis, MO, A1978).

Band densities were assessed using UN-SCAN-IT software (Silk Scientific, Orem, UT).

### qRT-PCR

Quantitative Real-Time PCR was performed using total RNA from lungs of E18.5 mice, and was conducted as previously described [18]. After isolation, total RNA was converted to cDNA, and qRT-PCR was performed using primers specific for *Rage* (5′- ACT ACC GAG TCC GAG TCT ACC -3′ and 5′- GTA GCT TCC CTC AGA CAC ACA −3′) and *GAPDH* (5′- TAT GTC GTG GAG TCT ACT GGT -3′ and 5′- GAG TTG TCA TAT TTC TCG TGG -3′) synthesized and HPLC purified by Invitrogen Life Technologies (Grand Island, NY).

### Gelatin zymography

Gelatin zymography was conduced to assess active MMP-9 expression in total lung protein. The activity of MMP-9 was examined by running samples on a polyacrylamide gel made with the addition of gelatin, which is a substrate of MMP-9. The enzymes were allowed to digest gelatin after sample loading and the gel was subsequently stained with Coomassie blue (0.25%) to detect the presence of MMP-9, which was observed in unstained regions of the gel where gelatin was decreased due to MMP-9 digestion.

### Cytokine characterization

Total lung lysates were obtained and quantified using the BCA technique. After quantification, total TNF-α and IL-1β levels were detected in 15 μg aliquots of total lung protein using specific ELISAs (Boster Biological Technology, Fremont, CA) as outlined in the provided manufacturer’s instructions. Groups were assessed in triplicate and statistical assessments were completed.

### Statistical analysis

Results are presented as the means ± S.D. of six replicate pools per group. Means were assessed by one and two-way analysis of variance (ANOVA). When ANOVA indicated significant differences, student t tests were used with Bonferroni correction for multiple comparisons. Results are representative and those with p values <0.05 were considered significant.

## Results

### Secondhand smoke (SHS) exposure during embryogenesis induces RAGE expression

**Figure 1:**
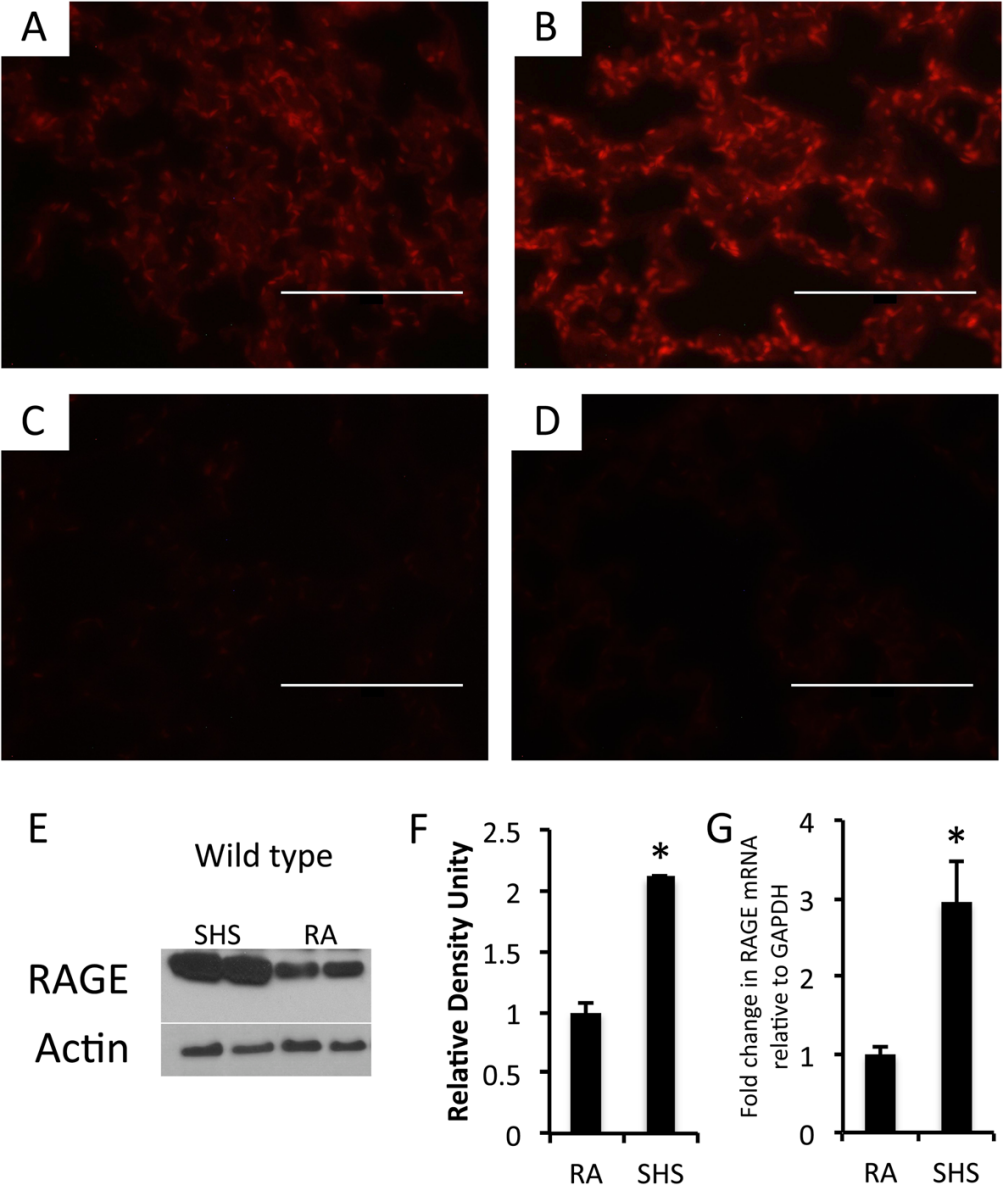
**Significant up-regulation of RAGE was observed by immunofluorescence using sections of adult mouse lung from wild type dams (A and B) and age-matched RAGE KO animals (C and D).** Specifically, red RAGE immunofluorescence was increased in wild type lungs exposed to SHS **(B)** compared to room air controls **(A)** and no expression was detected in RAGE KO animals exposed to room air **(C)** or SHS **(D)**. Images were at 100X original magnification and scale bars equal 100 nm. Immunoblotting using 10 μg of total lung protein revealed marked up-regulation of RAGE in lungs exposed to SHS compared to room air controls **(E)**. Densitometry of RAGE revealed an approximate 100% increase in protein expression **(F)** and quantitative RT-PCR revealed a significant up-regulation of RAGE mRNA **(G)**. Immunoblotting and qPCR data are representative of experiments performed in triplicate and statistical differences are noted (*P ≤0.05).

Pregnant dams were nasally exposed to SHS during the last four consecutive days of gestation. Compared to basal pulmonary RAGE expression (Figure 1A), immunofluorescence revealed that lungs in wild type pregnant dams respond to SHS exposure by increasing RAGE expression (Figure 1B). Confirmatory experiments that utilized immunoblotting were aimed at quantifying RAGE expression. Compared to room air exposed animals, lungs from pregnant wild type mice exposed to SHS markedly increased RAGE protein expression (Figure 1E) and densitometry of the bands suggested a significant increase (Figure 1F). RAGE expression was not detected in RAGE null mice following room air exposure (Figure 1C) or exposure to SHS (Figure 1D). Lastly, quantitative RT-PCR was performed using total RNA from adult lungs in order to correlate protein and mRNA expression levels. A significant increase in RAGE mRNA was observed in wild type animals exposed to SHS when compared to room air controls (Figure 1G).

**Figure 2:**
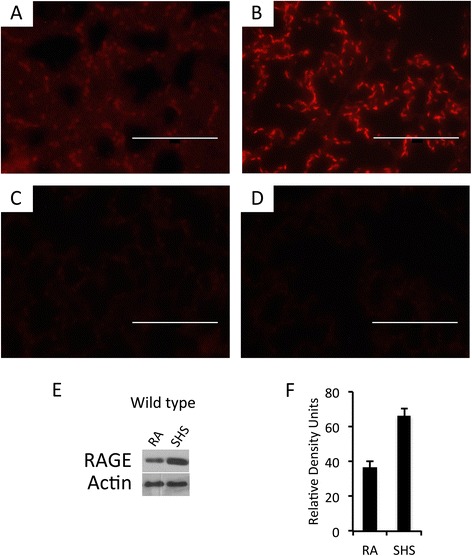
**Significant up-regulation of RAGE was observed by immunofluorescence using sections of E18.5 mouse lungs in pups from time mated pregnant wild type dams (A and B) and age-matched RAGE KO animals (C and D).** Specifically, red RAGE immunofluorescence was increased in wild type lungs exposed to SHS **(B)** compared to room air controls **(A)** and no expression was detected in RAGE KO animals exposed to room air **(C)** or SHS **(D)**. Images were at 100X original magnification and scale bars equal 100 nm. Immunoblotting using 10 μg of total E18.5 lung protein revealed marked up-regulation of RAGE in lungs exposed to SHS compared to room air controls **(E)**. Densitometry of RAGE in the blots revealed an approximate 100% increase in protein expression **(F)**. Immunoblotting data are representative of experiments performed in triplicate.

Immunofluorescence and immunoblotting for RAGE were next repeated using fetal E18.5 lung samples obtained from pups derived from room air or SHS-exposed pregnant dams. As was the case in pregnant dams, wild type pups experienced a notable increase in RAGE localization following SHS (Figure 2B) when compared to room air controls (Figure 2A). Immunblotting (Figure 2E) and densitometry of the resulting bands (Figure 2F) further confirmed an increase in RAGE expression as a product of SHS availability. Just as was observed in adults, RAGE was not detected in tissues obtained from RAGE KO animals (Figure 2C and D).

### RAGE abrogation protected against SHS-induced fetal weight loss and lung apoptosis

**Figure 3:**
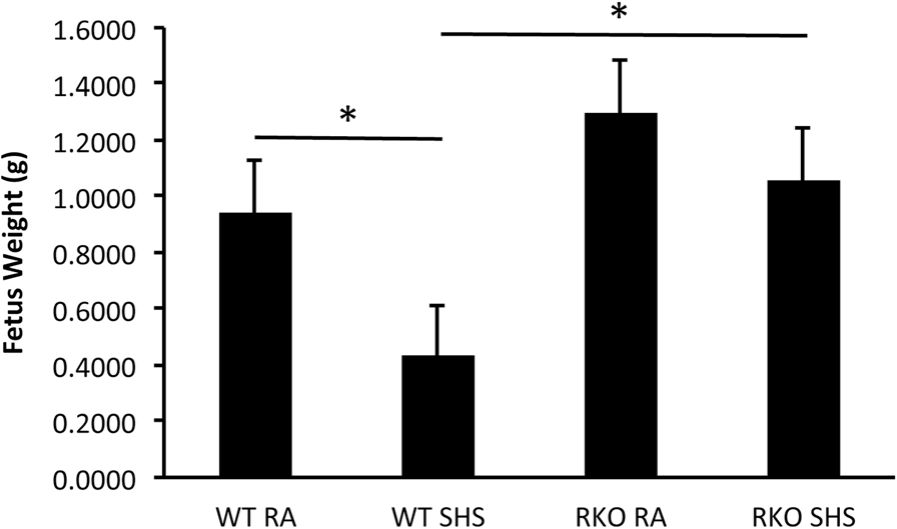
**Pups from SHS exposed dams were noticeably smaller.** Measurement of total body weights revealed that SHS-exposed wild type pups were significantly decreased compared to age-matched pups from dams exposed to room air during gestation. SHS-exposed RAGE KO pup weights were not significantly decreased when compared to age-matched RAGE KO pups exposed to room air. Furthermore, wild type pups + SHS animals were significantly smaller than RAGE KO + SHS animals. *Statistical difference (P ≤0.05) with at least nine pups per group.

**Figure 4:**
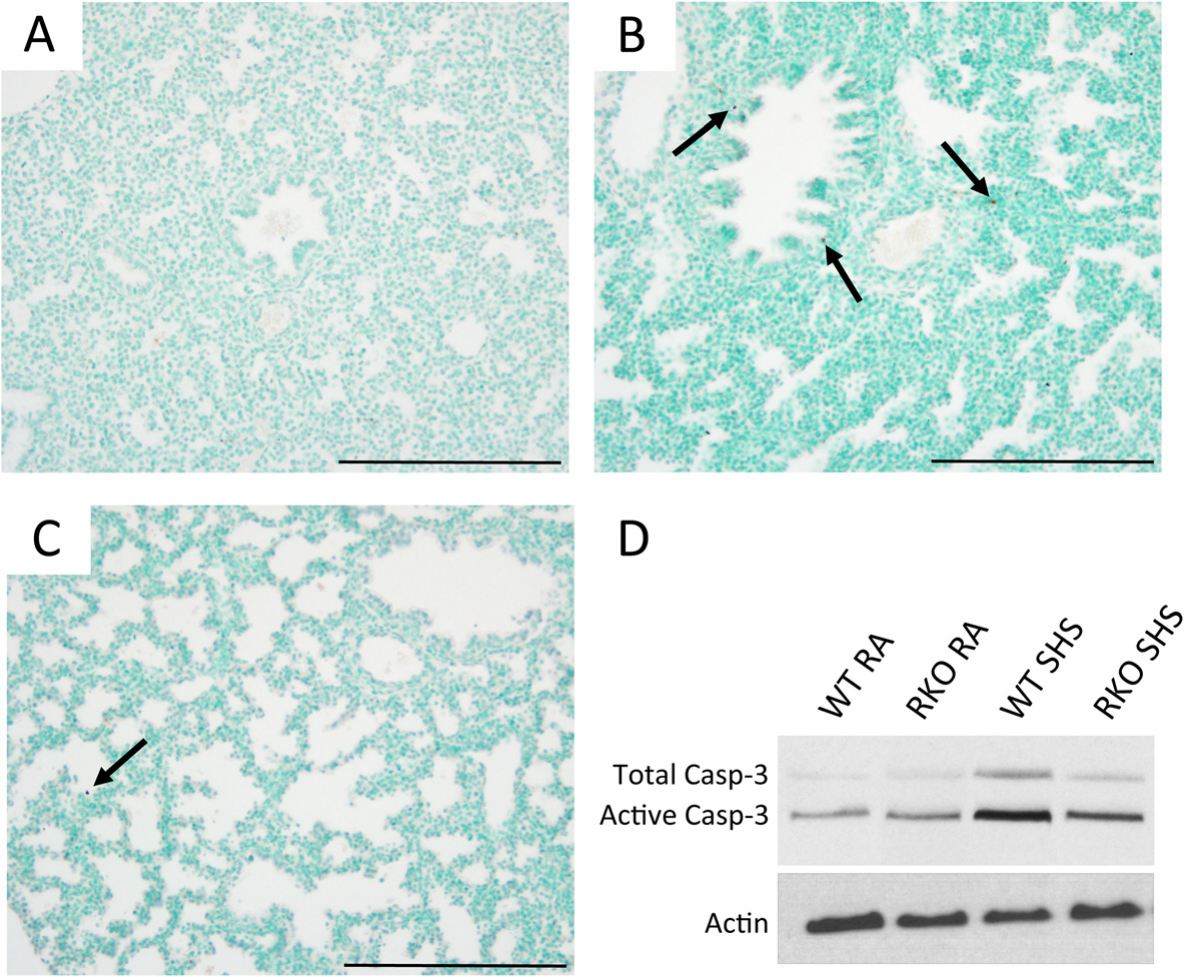
**A detectable increase in the number of actively apoptosing cells by TUNEL staining was observed in wild type pups exposed to SHS (arrows, B) when compared to room air controls (A).** There was no difference in the occurrence of TUNEL-positive cells when room air exposed wild type pups **(A)** and RAGE KO pups (not shown) were compared. Apoptotic cells were observed in RAGE KO pups exposed to SHS (**C**, arrow); however, the frequency of positive cells was markedly decreased. Blotting for caspase-3 revealed significantly more active caspase-3 in wild type pups exposed to SHS when compared to RAGE KO pups exposed to SHS **(D)**. Images were at 100X original magnification and scale bars equal 100 nm.

Fetal weights were obtained due to the observation that SHS exposure potentially correlated with smaller offspring. Average total fetal weights revealed that SHS-exposure of wild type dams resulted in a significant decrease (Figure 3). Averages also revealed that RAGE KO mice experienced no significant weight loss following SHS exposure when compared to room air exposed counterparts (Figure 3). Because of the link between RAGE signaling and apoptosis in the developing lung [6], we next assessed apoptotic trends via immunostaining for TdT-FragEL DNA Fragmentation (TUNEL), a common marker of cell death, and caspase-3. Apoptosis was not qualitatively detected by TUNEL staining in lungs from E18.5 mice whose mothers experienced room air throughout gestation (Figure 4A). TUNEL labeling revealed sporadic cells actively undergoing apoptosis in lungs obtained from SHS-exposed wild type mice (Figure 4B, arrows). While RAGE KO mice also manifested apoptotic cells following SHS exposure (Figure 4C), the frequency of TUNEL positive cells was detectibly diminished. Immunoblotting for active caspase-3 revealed increased expression following SHS exposure; however, active caspase-3 was decreased in SHS-exposed RAGE KO pups compared to wild type + SHS pups (Figure 4D).

### RAGE mediates diminished collagen deposition and elevated MMP-9 expression following SHS exposure

**Figure 5:**
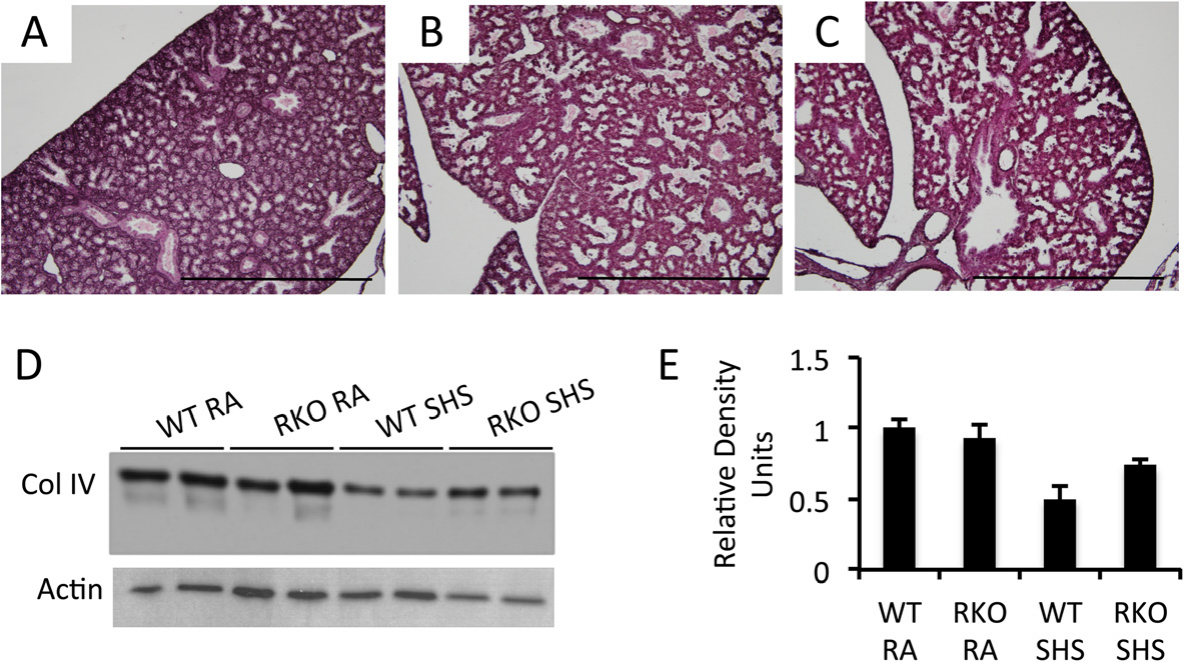
**SHS exposure decreases collagen IV expression and RAGE KO mice lessen the degree of SHS-induced changes in collagen IV expression.** Lungs from wild type pups exposed to room air **(A)**, SHS-exposed wild type **(B)**, and SHS-exposed RAGE KO lungs **(C)** were stained for type IV collagen to qualitatively determine relative changes in type IV collagen associated with basement membranes. Only subtly qualitative decreases in type IV collagen were observed in SHS exposed animals. Immunoblotting for type IV collagen using equal aliquots of 10 μg total lung protein revealed markedly decreased total type IV collagen expression in SHS-exposed pups **(D)**. Immunoblotting **(D)** and densitometry of the resulting bands **(E)** demonstrated that SHS-induced decreases in type IV collagen expression in wild type animals is more severe when compared to SHS-exposed RAGE KO mice. Immunoblotting data are representative of experiments performed in triplicate. Images were at 100X original magnification and scale bars equal 100 nm.

An immunohistochemical analysis of type IV collagen was completed on lungs from pups obtained from room air and SHS-exposed dams. The relative abundance of type IV collagen was sought due to it being a plentiful collagen subtype common in basement membranes. Staining for type IV collagen revealed a qualitative decrease in lungs from wild type mice exposed to SHS (Figure 5B) compared to room air exposed controls (Figure 5A). Lungs from SHS-exposed RAGE KO mice also appeared to have diminished type IV collagen abundance (Figure 5C). Quantification of total type IV collagen was obtained by immunoblotting and subsequent densitometry revealed that SHS exposure correlated with decreased expression (Figure 5D and E). Interestingly, decreased type IV collagen synthesis following SHS exposure was partially blunted in pups from RAGE KO animals (Figure 5D and E).

**Figure 6:**
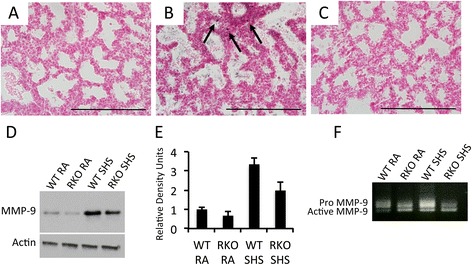
**SHS exposure increases MMP-9 expression and RAGE KO mice lessen the degree of SHS-mediated MMP-9 augmentation.** Lungs from wild type room air exposed **(A)**, SHS-exposed wild type **(B)**, and SHS-exposed RAGE KO mice **(C)** were qualitatively immunostained for MMP-9. SHS induced a detectibly higher number of MMP-9 positive cells (B, arrows) compared to SHS-exposed RAGE KO mice **(C)**. Immunoblotting for MMP-9 **(D)** and related densitometry **(E)** using standardized aliquots of 10 μg total lung protein revealed markedly increased MMP-9 expression levels in SHS-exposed wild type animals and notable protection in SHS-exposed RAGE KO animals. Gelatin zymography revealed increased synthesis of MMP-9 by SHS-exposed wild type animals compared to all other groups and active MMP-9 was also significantly elevated in SHS-exposed wild type animals compared to SHS-exposed RAGE KO mice **(F)**. Immunoblotting data are representative of experiments performed in triplicate. Images were at 100X original magnification and scale bars equal 100 nm.

**Figure 7:**
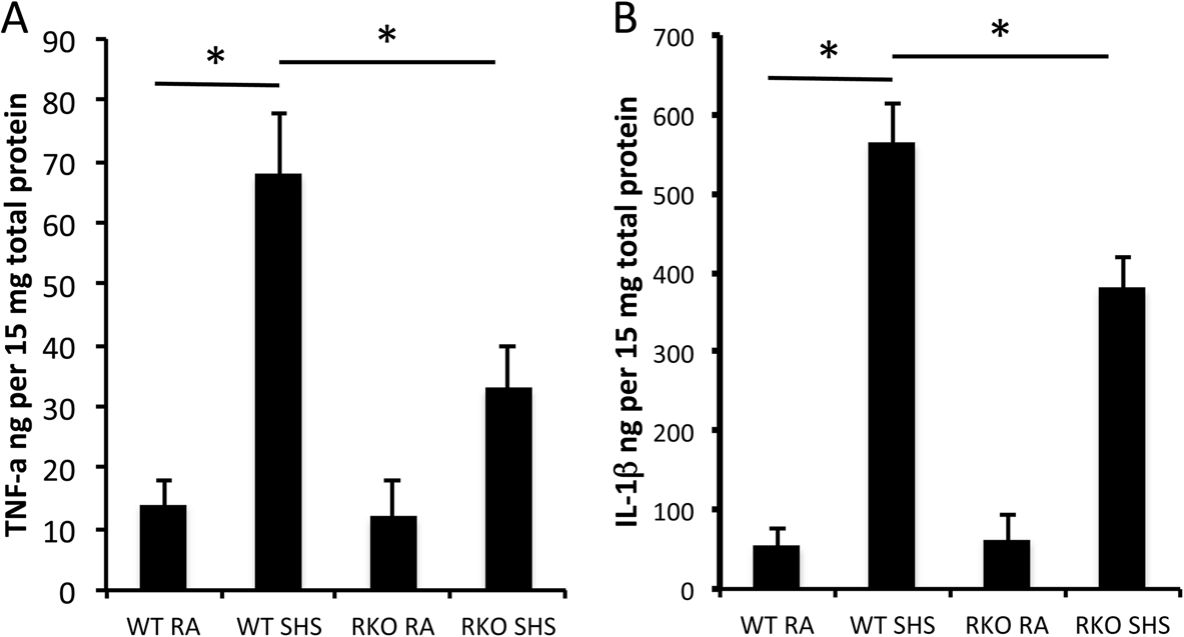
**Pro-inflammatory cytokine expression was assessed per 15 μg total lung protein obtained from E18.5 pups by ELISA.** TNF-α **(A)** and IL-1β **(B)** levels were both significantly increased in SHS-exposed wild type pup lungs compared to room air exposed controls. Cytokine levels were not different in lungs from wild type and RAGE KO mice exposed to room air. Lung samples from SHS-exposed RAGE KO pups revealed significant decreases in SHS-induced cytokine expression when compared to SHS-exposed controls. *Statistical differences (P ≤0.05) with at least three replicates per group.

In order to mechanistically identify molecules that potentially function in the targeting of collagen metabolism, an analysis of matrix metalloprotease 9 (MMP-9), TNF-α, and IL-1β was conducted. MMP-9 is a downstream effector of RAGE and it degrades a host of collagens, including type IV [19,20]. Immunostaining for MMP-9 revealed a marked increase in pulmonary cells obtained from E18.5 wild type pups following SHS (Figure 6B) when compared to room air exposed controls (Figure 6A). While MMP-9 positive cells were also observed in lung sections from SHS-exposed RAGE KO mice (Figure 6C), their frequency was reduced. Immunoblotting was next employed in order to quantitatively assess MMP-9 expression in total lung homogenates. Blotting (Figure 6D) and densitometry (Figure 6E) suggested profound up-regulation of MMP-9 in SHS-exposed wild type mice. However, the absence of RAGE, at least in part, protected SHS-exposed RAGE KO mice from the high MMP-9 levels observed in SHS-exposed wild type mice (Figure 6D and E). Pro and active forms of MMP-9 were also evaluated and data suggest enhanced synthesis and greater activation in wild type animals exposed to SHS compared to RAGE KO mice exposed to SHS (Figure 6F). TNF-α and IL-1β are pro-inflammatory mediators implicated in diverse inflammatory conditions. As anticipated, TNF-α, and IL-1β were both significantly increased in the lungs of SHS-exposed control pups when compared to room air controls (Figure 7A and B). Furthermore, pulmonary expression of TNF-α and IL-1β was blunted in SHS-exposed RAGE null pups when compared to SHS-exposed controls (Figure 7A and B).

## Discussion and conclusions

### Effects of SHS on RAGE expression and fetal weight

The current investigation sought to simultaneously elucidate the expression profiles of pulmonary RAGE in gestating dams and their developing fetuses following SHS exposure. To our knowledge, this is the first study that seeks to delineate the biology of RAGE in the context of SHS exposure. Nicotine and a host of other tobacco related entities are known to traverse the placental barrier [21] and lungs of such exposed fetuses experience developmental anomalies including branching defects and cell differentiation delays [22]. Our discovery that dams and pups both induce higher RAGE expression suggests potential RAGE-mediated coping mechanisms that clearly warrant future exhaustive studies. Unequivocal support for deleterious inflammation mediated by tobacco smoke-induced RAGE has been provided by our laboratory [3,18,23,24]. While the main intent of the current publication was not to delineate the inflammatory effects of RAGE signaling during SHS exposure or the specific contributions of its participants, its augmentation alone necessitates concern. In fact, a higher level of concern is suggested by this and related work due to the fact that the dams in the current study were exposed to SHS. Research performed by Seller and Bnait [25] and several others was conducted on the premise that dams exposed to primary smoking may confer second hand effects in pups including skeletal abnormalities and neural tube defects. Our research indicates that in addition to primary smokers that are unable or unwilling to quit, even SHS exposure of expecting adults is a concern for the developing fetus.

The observation that fetal weights were decreased following SHS exposure supports similar findings from previous work using primary smoking [26]. An unanticipated, yet highly intriguing result of our studies was the effect RAGE inhibition had on SHS stimulation. Smaller gestational and term weights are associated with a host of perinatal complications related to renal development, cardiovascular efficiencies, and sensory processing [27,28]. Because RAGE knock out pups exposed to SHS were not smaller at term, further inspection into potential mechanisms of organismal health protection would be highly informative. As an initial inspection, we have recently discovered increased RAGE expression in human and mouse placentae following smoke exposure. Additional studies should consider RAGE-mediated effects on trophoblast invasion/viability, nutrition, and possible pulmonary sources of AGE-induced inflammatory cytokines such as TNF-α that systemically compromise the organism.

### Effects of SHS and RAGE on pulmonary matrix deposition

SHS-induced alterations in cell turnover, including instances of apoptosis, are complicated by compromised extracellular matrix. Cells necessary for the normal physiology of the lung such as endothelium, respiratory epithelium, and conducting airway epithelium require spatial integrity stabilized by collagen and other matrix molecules. A significant determinant of the architectural matrix between cells is due to type collagen IV synthesis and secretion [29,30]. In fact, the importance of type IV collagen as a stabilizing molecule is confirmed in research centering on COPD [31] and other adult inflammatory diseases [32]. Our qualitative and quantitative data revealing diminished type IV collagen identifies an important concept relating to SHS exposure. First of all, extracellular matrixes are targeted by smoke exposure and secondly, RAGE expressed by epithelial cells plausibly functions to signal and regulate cells that secrete matrix in the developing lung.

RAGE-ligand interactions initiate cellular communication via the activation of signaling intermediates prior to the activation of NF-κB [24]. The current research sought to determine to what extent MMP-9, a matrix metalloprotease (MMP) secreted by fibroblasts, alveolar macrophages, and epithelial cells, functions in the SHS exposed pups. MMPs are endopeptidases that can destroy components of the extracellular matrix and MMP-9 is an NF-κB target [33] that specifically targets type IV collagen [34]. Because of their destructive capabilities, MMPs are recognized as not only central players in cases of disease, but during remodeling events observed during development as well [35]. For example, MMPs are also considered potent effectors of normal lung morphogenesis that assist in the orchestration of definitive lung parenchyma [36,37]. Notably, MMP-9 has been directly implicated in the progression of bronchopulmonary dysplasia (BPD), a developmental anomaly characterized by inflammation, lack of alveolar septation, and abnormal pulmonary vascular development [38]. Known for perpetuating inflammatory axes, TNF-α and IL-1β induce the release of numerous inflammatory cytokines, enhance leukocyte adhesion during chemotactic transmigration, and coordinate MMP-9 elaboration [39-41]. Interestingly, mouse models of inflammatory diseases have demonstrated a link between the availability of TNF-α and IL-1β and the direct effects of MMP-9 on cell survival, inflammation status, and matrix durability [42]. Our work builds upon these discoveries by identifying MMP-9 as a SHS target that likely effectuates end points via RAGE-mediated pathways.

In summary, RAGE expression and matrix destabilization are probable byproducts of pulmonary SHS exposure during embryogenesis. This study suggests that protection from damaging SHS-induced effects such as fetal weight decreases, matrix abundance, and MMP imbalances is possible when RAGE is inhibited. Despite notable advancements in SHS research provided by the current research, additional work is still necessary that focuses on RAGE signaling during pulmonary branching morphogenesis and to what extent RAGE alone is capable of inducing SHS related lung phenotypes.

## References

[1] Ten Have-Opbroek AA (1991). Lung development in the mouse embryo. Exp Lung Res.

[2] Buckley ST, Ehrhardt C (2010). The receptor for advanced glycation end products (RAGE) and the lung. J Biomed Biotechnol.

[3] Reynolds PR, Kasteller S, Cosio MG, Sturrock A, Huecksteadt TP, Hoidal JR (2008). RAGE: developmental expression and positive feedback regulation by Egr-1 during cigarette smoke exposure in pulmonary epithelial cells. Am J Physiol Lung Cell Mol Physiol.

[4] Ott C, Jacobs K, Haucke E, Navarrete Santos A, Grune T, Simm A (2014). Role of advanced glycation end products in cellular signaling. Redox Biol.

[5] Demling N, Ehrhardt C, Kasper M, Laue M, Knels L, Rieber EP (2006). Promotion of cell adherence and spreading: a novel function of RAGE, the highly selective differentiation marker of human alveolar epithelial type I cells. Cell Tissue Res.

[6] Stogsdill JA, Stogsdill MP, Porter JL, Hancock JM, Robinson AB, Reynolds PR (2012). Embryonic over-expression of RAGE by alveolar epithelium induces an imbalance between proliferation and apoptosis. Am J Respir Cell Mol Biol.

[7] Yamagishi S, Matsui T, Nakamura K (2008). Possible involvement of tobacco-derived advanced glycation end products (AGEs) in an increased risk for developing cancers and cardiovascular disease in former smokers. Med Hypotheses.

[8] Taguchi A, Blood DC, del Toro G, Canet A, Lee DC, Qu W, Tanji N, Lu Y, Lalla E, Fu C, Hoffman MA, Kislinger T, Ingram M, Lu A, Tanaka H, Hori O, Ogawa S, Stern DM, Schmidt AM (2000). Blockade of RAGE-amphoterin signalling suppresses tumour growth and metastases. Nature.

[9] Reynolds PR, Kasteler SD, Schmitt RE, Hoidal JR (2010). RAGE signals through ras during tobacco smoke-induced pulmonary inflammation. Am J Resp Cell Mol Biol.

[10] Bianchi R, Giambanco I, Donato R (2010). S100B/RAGE-dependent activation of microglia via NF-κB and AP-1 co-regulation of COX-2 expression by S100B, IL-1β and TNF-α. Neurobiol Aging.

[11] Rycroft CE, Heyes A, Lanza L, Becker K (2012). Epidemiology of chronic obstructive pulmonary disease: a literature review. Int J Chron Obstruct Pulmon Dis.

[12] Tager IB, Hanrahan JP, Tosteson TD, Castile RG, Brown RW, Weiss ST, Speizer FE (1993). Lung function, pre- and post-natal smoke exposure, and wheezing in the first year of life. Am Rev Respir Dis.

[13] Rinaldi M, Maes K, De Vleeschauwer S, Thomas D, Verbeken EK, Decramer M, Gayan-Ramirez GN (2012). Long-term nose-only cigarette smoke exposure induces emphysema and mild skeletal muscle dysfunction in mice. Dis Model Mech.

[14] Vlahos R, Bozinovski S, Chan SP, Ivanov S, Linden A, Hamilton JA, Anderson GP (2010). Neutralizing granulocyte/macrophage colony-stimulating factor inhibits cigarette smoke-induced lung inflammation. Am J Respir Crit Care Med.

[15] Reynolds PR, Mucenski ML, LeCras TD, Nichols WC, Whitsett JA (2004). Midkine (MK) induces myocardin during hypoxia and causes pulmonary vascular remodeling. J Biol Chem.

[16] Reynolds PR, Mucenski ML, Whitsett JA (2003). Thyroid Transcription Factor (TTF)-1 regulates the expression of Midkine (MK) during lung morphogenesis. Dev Dyn.

[17] Reynolds PR, Kasteler SD, Sturrock A, Sanders K, Kennedy TP, Hoidal JR (2009). RAGE targeting protects against hyperoxia-induced lung injury in mice. Am J Resp Cell Mol Biol.

[18] Robinson AB, Johnson KD, Bennion BG, Reynolds PR (2012). RAGE signaling by alveolar macrophages influences tobacco smoke-induced inflammation. Am J Physiol Lung Cell Mol Physiol.

[19] Parks WC, Shapiro SD (2001). Matrix metalloproteinases in lung biology. Respir Res.

[20] Stogsdill MP, Stogsdill JA, Bodine BG, Fredrickson AC, Sefcik TL, Wood TA, Kasteler SK, Reynolds PR (2013). Conditional RAGE over expression in the adult murine lung causes airspace enlargement and induces inflammation. Am J Resp Cell Mol Biol.

[21] Vaglenova J, Birru S, Pandiella NM, Breese CR (2004). An assessment of the long-term developmental and behavioral teratogenicity of prenatal nicotine exposure. Behav Brain Res.

[22] Sekhon HS, Jia Y, Raab R, Kuryatov A, Pankow JF, Whitsett JA, Spindel ER (1999). Prenatal nicotine increases pulmonary α7 nicotinic receptor expression and alters fetal lung development in monkeys. J Clin Invest.

[23] Reynolds PR, Hoidal JR (2006). Cigarette smoke-induced Egr-1 upregulates pro-inflammatory cytokines in pulmonary epithelial cells. Am J Resp Cell Mol Biol.

[24] Robinson AB, Stogsdill JA, Lewis JP, Wood TT, Reynolds PR (2012). RAGE and tobacco smoke: insights into modeling chronic obstructive pulmonary disease. Front Physiol.

[25] Seller MJ, Bnait KS (1995). Effects of tobacco smoke inhalation on the developing mouse embryo and fetus. Reprod Toxicol.

[26] Esposito ER, Horn KH, Greene RM, Pisano MM (2008). An animal model of cigarette smoke-induced in utero growth retardation. Toxicology.

[27] Gill SV, May-Benson TA, Teasdale A, Munsell EG (2013). Birth and developmental correlates of birth weight in a sample of children with potential sensory processing disorder. BMC Pediatr.

[28] Zohdi V, Sutherland MR, Lim K, Gubhaju L, Zimanyi MA, Black MJ (2012). Low birth weight due to intrauterine growth restriction and/or preterm birth: effects on nephron number and long-term renal health. Int J Nephrol.

[29] Nakano KY, Iyama KI, Mori T, Yoshioka M, Hiraoka T, Sado Y, Ninomiya Y (2001). Loss of alveolar basement membrane type IV collagen alpha3, alpha4, and alpha5 chains in bronchioloalveolar carcinoma of the lung. J Pathol.

[30] Timpl R (1989). Structure and biological activity of basement membrane proteins. Eur J Biochem.

[31] Atkinson JJ, Senior RM (2003). Matrix metalloproteinase-9 in lung remodeling. Am J Respir Cell Mol Biol.

[32] Ohbayashi H, Shimokata K (2005). Matrix metalloproteinase-9 and airway remodeling in asthma. Curr Drug Targets Inflamm Allergy.

[33] Soler-Cataluna JJ, Martinez-Garcia MA, Roman Sanchez P, Salcedo E, Navarro M, Ochando R (2005). Severe acute exacerbations and mortality in patients with chronic obstructive pulmonary disease. Thorax.

[34] Pardo A, Selman M (2006). Matrix metalloproteases in aberrant fibrotic tissue remodeling. Proc Am Thorac Soc.

[35] Page-McCaw A, Ewald AJ, Werb Z (2007). Matrix metalloproteinases and the regulation of tissue remodeling. Nat Rev Mol Cell Biol.

[36] Lee Y, Fryer JD, Kang H, Crespo-Barreto J, Bowman AB, Gao Y, Kahle JJ, Hong JS, Kheradmand F, Orr HT, Finegold MJ, Zoghbi HY (2011). ATXN1 protein family and CIC regulate extracellular metrix remodeling and lung alveolarization. Dev Cell.

[37] Buckley ST, Medina C, Kasper M, Ehrhardt C (2011). Interplay between RAGE, CD44, nd focal adhesion molecules in epithelial-mesenchymal transition of alveolar epithelial cells. Am J Physiol Lung Cell Mol Physiol.

[38] Clsno JJ (2003). Pathology of new bronchopulmonary dysplasia. Semin Neonatol.

[39] Laskin DL, Sunil VR, Fakhrzadeh L, Groves A, Gow AJ, Laskin JD (2010). Macrophages, reactive nitrogen species, and lung injury. Ann N Y Acad Sci.

[40] Kyan-Aung U, Haskard DO, Poston RN, Thornhill MH, Lee TH (1991). Endothelial leukocyte adhesion molecule-1 and intercellular adhesion molecule-1 mediate the adhesion of eosinophils to endothelial cells in vitro and are expressed by endothelium in allergic cutaneous inflammation in vivo. J Immunol.

[41] Ryder MI, Saghizadeh M, Ding Y, Nguyen N, Soskolne A (2002). Effects of tobacco smoke on the secretion of interleukin-1beta, tumor necrosis factor-alpha, and transforming growth factor-beta from peripheral blood mononuclear cells. Oral Microbiol Immunol.

[42] Xue M, McKelvey K, Shen K, Minhas N, March L, Park SY, Jackson CJ: **Endogenous MMP-9 and not MMP-2 promotes rheumatoid synovial fibroblast survival, inflammation, and cartilage degradation.** *Rheumatology (Oxford)* 2014: June 29, Epub doi:10.1093/rheumatology/keu254 10.1093/rheumatology/keu254 24982240

